# Synthesis of Highly Energetic PolyNitrogen by Nanosecond-Pulsed Plasma in Liquid Nitrogen

**DOI:** 10.3390/ma14154292

**Published:** 2021-07-31

**Authors:** Danil Dobrynin, Zhiheng Song, Alexander Fridman

**Affiliations:** C&J Nyheim Plasma Institute, Drexel University, 200 Federal Street, Suite 500, Camden, NJ 08103, USA; zs344@drexel.edu (Z.S.); af55@drexel.edu (A.F.)

**Keywords:** nanosecond plasma in liquid, polymeric nitrogen, energetic materials

## Abstract

We report on an experimental study of nanosecond-pulsed plasma treatment of liquid nitrogen demonstrating synthesis of a highly energetic nitrogen material. Raman, FTIR analysis of gas phase products of decomposition, and the material explosion characteristics suggest synthesis of polymeric (amorphous) nitrogen compound which is stable at ambient pressure up to temperatures of about −150 °C. Addition of adsorbents with relatively large characteristic pore sizes (>5 nm) allows marginally improved recovery of the material as determined by temperature-dependent Raman measurements. By analyzing the shock wave propagation resulting from the explosions, we estimated the energy density of the material to be 13.3 ± 3.5 kJ/g, close to the previously predicted value for amorphous polymeric nitrogen.

## 1. Introduction

Nitrogen-rich compounds that contain single- and double-bonded nitrogen offer potential as high-energy density materials [1,2,3]. Transformation of nitrogen in these compounds into triple-bonded nitrogen molecules is followed by a large energy release. Theoretical studies have predicted energy densities for all-nitrogen polymeric network of ~11.3 kJ/g [4], making it more powerful than one of the most commonly known explosive chemicals—trinitrotoluene (TNT, 4.1 kJ/g) and azidoazide azide (6.8 kJ/g), an unstable compound containing 14 nitrogen atoms [5]. However, the synthesis of polynitrogen materials is challenging, and it is typically produced by the transformation of molecular nitrogen at high (tens of GPa) pressures (see, for example, [6,7]).

In our recent studies, we have employed nanosecond-pulsed plasmas ignited in liquid nitrogen as a tool for production and fast quenching of nitrogen-rich materials [8,9]. Liquid nitrogen plasmas, with their relatively high energies, pressures, radiation, and the high densities of reactive species, are suggested to be an effective way to synthesize new materials [10,11,12,13,14,15]. Here we report on the observation of generation of energetic nitrogen-based material by nanosecond-pulsed spark plasma in liquid nitrogen. We show that plasma treatment results in the production of amorphous nitrogen that can be adsorbed by materials with characteristic pore sizes of >5 nm. The explosion of the adsorbed material was studied using high-speed imaging for estimation of energy density.

## 2. Materials and Methods

For the generation of a spark discharge in liquid nitrogen, two stainless-steel needles with ~100 μm tip curvature were fixed with a ~1 mm gap in a double-walled glass (560 mL) chamber covered with a vented lid. High voltage pulses were generated using FPG 20-1NM10DD high voltage plasma source (FID Tech Company, Burbach, Germany) capable of providing pulses with maximum amplitude of 26.8 kV, rise time (10–90% amplitude) of 3–4 ns and duration (63% amplitude) of 11.6 ns. High voltage pulses were delivered to the electrodes via 3 m long RG 393/U 50 Ohm high voltage coaxial cable.

Industrial grade (99.999% N_2_, O_2_ ≤ 5 ppm, CO_2_ ≤ 10 ppm) liquid nitrogen in all of the experiments was purchased from Airgas, USA. In Table 1, a list of the adsorbents used in the study and their characteristics are shown. In the experiments, the adsorbent materials (50 mg) were added to the liquid nitrogen either before or after the plasma treatment. In all cases, treatment was done using 20 kV amplitude pulses at 1 kHz repetition rate for 30 min.

Princeton Instruments—Acton Research TriVista TR555 spectrometer system and Princeton Instruments PIMAX ICCD camera in combination with SDM532-100SM-L 532 nm Spectrum Stabilized Laser Module (Newport, Irvine, CA, USA) were used for the measurements of Raman spectra. For that, excitation fiber of a RPB532 Raman probe (InPhotonics, Norwood, MA, USA) was connected to the laser source and emission fiber to the entrance slit of the spectrometer. The Raman probe was positioned at ~7.5 mm (focal length of the probe) above the examined samples. At the focal point, the probe spot size is approximately 160 μm and depth of field is ~2.2 mm. Spectra were typically recorded with 1 s exposure time and 10 accumulations. The spectrometer was calibrated using a 6035 Hg(Ar) calibration lamp (Newport, USA). The Raman spectra were registered from the treated samples directly in liquid nitrogen with a liquid layer up to a few mm in thickness (in low form Dewar flask, CG-1592-03, Chemglass Life Sciences, Vineland, NJ, USA). Raman spectra of the slowly heated sample were obtained in room air:treated samples were placed in a heavy (~500 g) metal sample holder to allow for a slower warm-up rate.

FTIR measurements were performed using a Nicolet 8700 FTIR spectrometer equipped with a 2 m gas cell with KBr windows, 200 mL internal volume (Thermo Fisher Scientific, Waltham, MA, USA), with 4 cm^−1^ spectral resolution.

Sample explosion shadow imaging was done using Miro M310 (Phantom, Wayne, NJ, USA) high-speed camera (15–30 μs exposure time; frame rates of 15,000–20,000 fps; final image resolution 256 × 256 pixels) and 30 W Deuterium arc lamp (Newport, USA) as a source of back light. Sample weight measurements were done using Fisher Scientific Accu-64 analytical balance (readability 0.1 mg).

## 3. Results and Discussion

**Figure 1:**
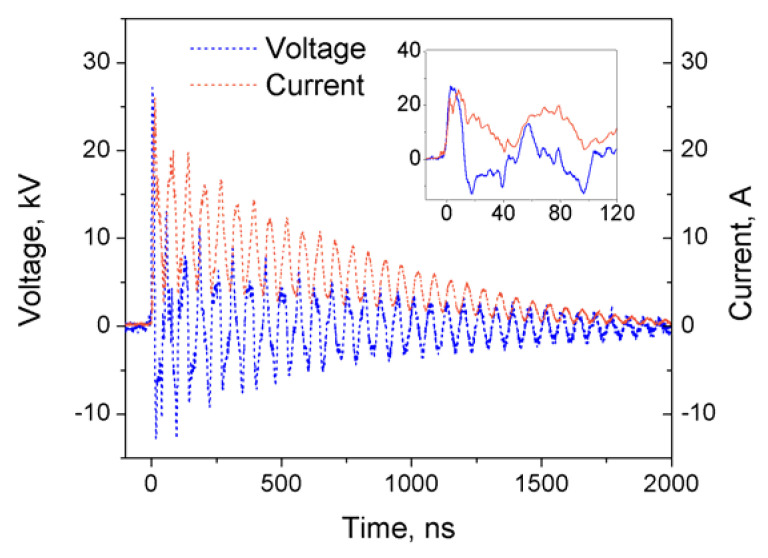
Current and voltage waveforms of spark discharge plasma ignited in liquid nitrogen.

In this study, we have used a nanosecond-pulsed spark discharge plasma for the treatment of liquid nitrogen. Here, we have used a relatively short coaxial high voltage cable which acts as a capacitive element (~300 pF capacitance); this results in the continuous re-ignition of hot nanosecond sparks during the characteristic pulse—as can be seen from the measured voltage and current waveforms shown in Figure 1—with a total deposited energy of ~36 mJ per event. These pulses were supplied to the discharge chamber at frequency of 1 kHz for 30 min, resulting in a total energy deposition of ~65 kJ. It must be mentioned that the thermal nature of the discharge results in a noticeable electrode erosion: after 30 min of treatment, the positive high voltage electrode loses about 0.2 mg (0.3% of initial weight), while the weight of the grounded needle is reduced by ~1.2 mg (2%).

**Figure 2:**
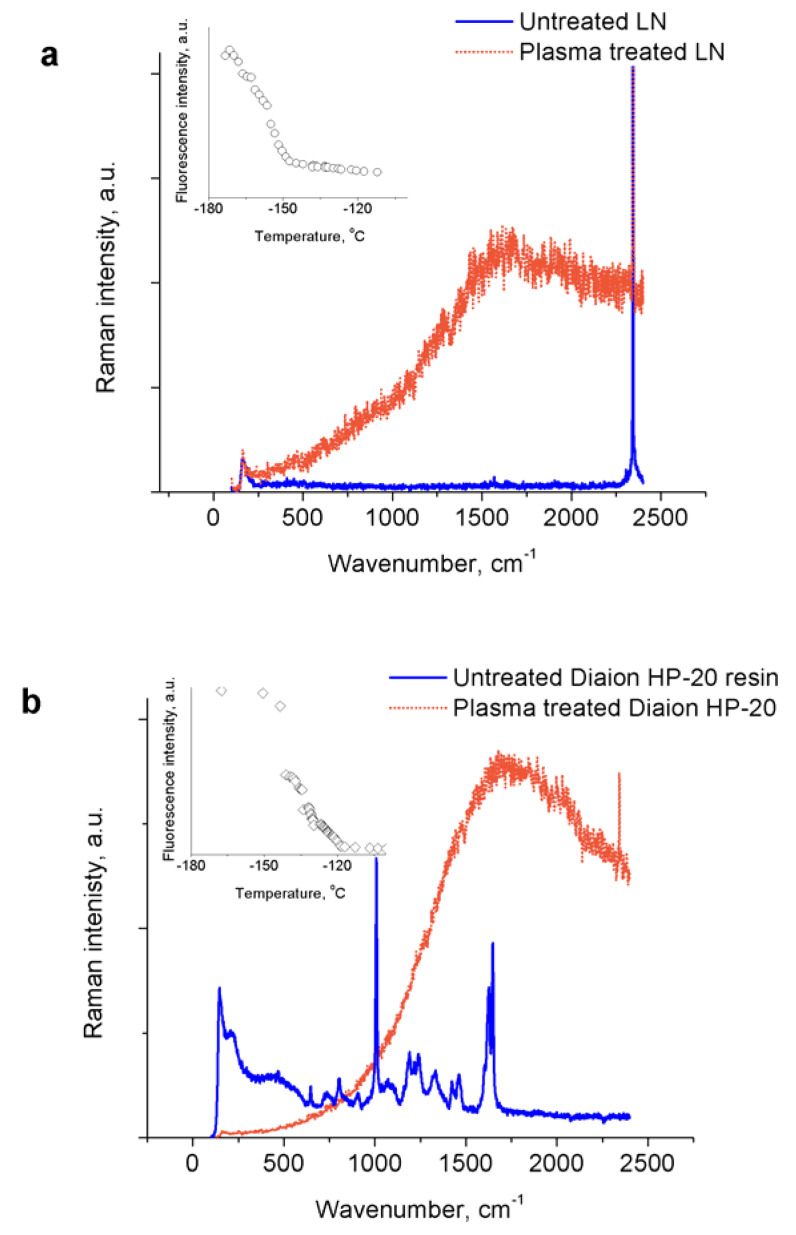
Raman spectra of untreated liquid nitrogen (LN) (**a**) and Diaion HP-20 resin (**b**) and treated with nanosecond-pulsed spark plasma. Inserts show decrease of the overall fluorescence intensity with increase of the sample temperature after evaporation of liquid nitrogen (linear scales). Peak around 2330 cm^−1^ originates from molecular nitrogen.

During the treatment, a continuous generation of black microparticles that typically deposit on the bottom of the glass chamber was observed. Upon rapid evaporation of the liquid, these particles condense leaving an explosive (if heated) black mass; however, explosion happens inconsistently and with a varying degree of intensity. We have made numerous attempts to obtain a Raman spectrum of the material generated in liquid nitrogen by the plasma—see Figure 2a—and in contrast to our previous study, where liquid nitrogen was treated by non-thermal corona-type plasma, no obvious new peaks were recorded. Instead, the sample typically exhibits a strong fluorescence signal from the plasma-generated microparticles, which disappears at around −150 °C (see insert in Figure 2a). Similar Raman spectra (sample fluorescence) was reported previously for high-pressure produced amorphous polynitrogen [16].

In an attempt to stabilize the plasma-produced material, we have performed a series of experiments in which various adsorbents (50 mg) were introduced either before or after the treatment. Right after the treatment—or after 1–24 h of incubation in the experiments when the adsorbent was added after the treatment—we monitored the explosivity of the samples upon rapid heating. Only the samples that contained adsorbents with larger average pore size—Diaion HP-2MG, Diaion HP-20 and activated carbon—have exploded. Treatment (or incubation) of the adsorbent resins has also resulted in their color change from white to dark (black). The Raman spectra of the treated Diaion HP-20 resin are shown in Figure 2b; as in the case of pure liquid nitrogen treatment, we did not register any new Raman peaks, but strong fluorescence which starts to diminish at temperature around −150 °C and disappears at around −120 °C (see insert in Figure 2b). This could indicate marginally improved stability of the physisorbed material. We failed to obtain Raman spectra from the treated activated carbon—visibly fluorescent particles escape and their fluorescence fades away (with flashes) under the laser beam, which indicates the highly unstable nature of the adsorbed material.

**Figure 3:**
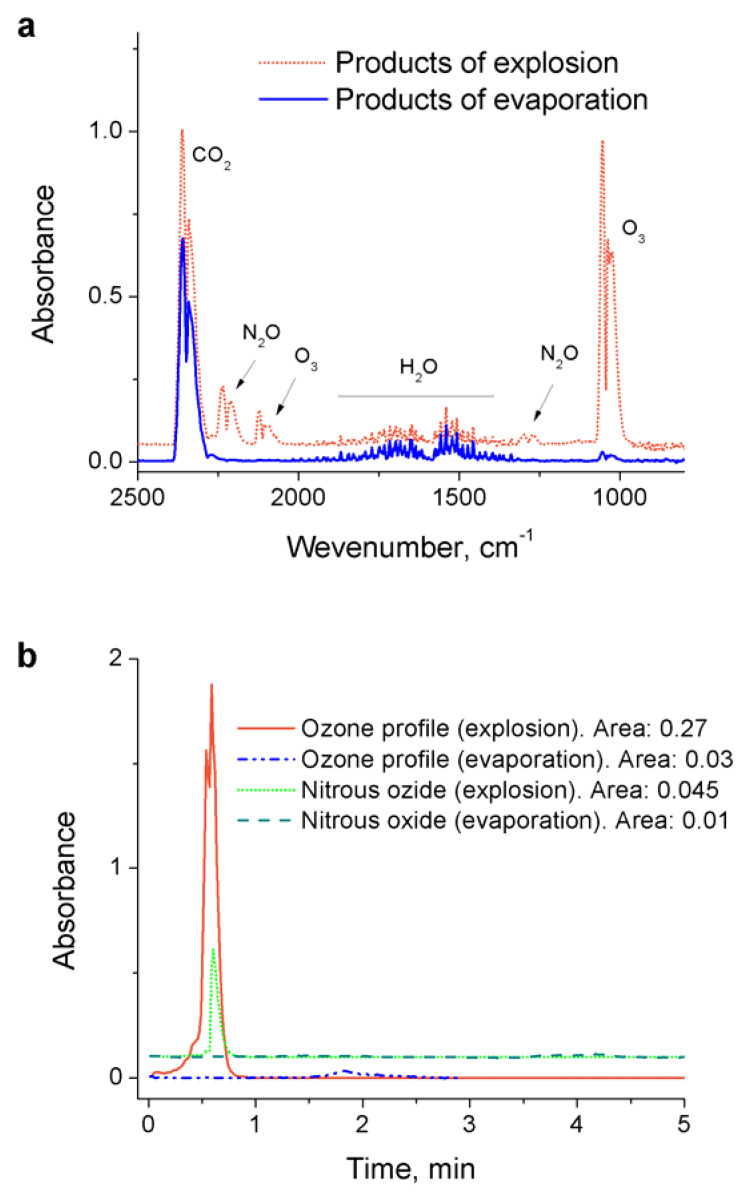
(**a**) FTIR spectra of gaseous products of the plasma-produced material (treated with activated carbon) evaporation and explosion in air; (**b**) time evolution of the ozone (1050 cm^−1^) and nitrous oxide (2235 cm^−1^) peaks.

FTIR analysis of the gaseous products of the samples treated in the presence of adsorbents evaporation and explosion in air was performed using the Nicolet 8700 FTIR spectrometer equipped with a 2 m gas cell. The samples were placed in a metal dish open to room air and the reaction products were collected into the gas cell via a gas flow created by a vacuum pump connected to the outlet of the cell (flow rate ~4 standard liters per minute). The representative spectra are shown in Figure 3.

FTIR spectra of the gaseous products from all samples indicate presence of ozone, nitrous oxide, water, and carbon dioxide. Evaporated samples show significantly lower concentrations of O_3_ and N_2_O: carbon dioxide is due to its presence in liquid nitrogen and contamination from room air, while ozone and nitrous oxide can be generated in liquid nitrogen from the oxygen impurities that are present in the untreated liquid nitrogen and contamination during the treatment container filling. It is, however, unlikely that ozone and nitrous oxide are generated directly by the discharge in liquid nitrogen: no other NO_x_ species have been detected (e.g., NO, NO_2_, N_2_O_5_) while they are usually produced in air plasmas in higher concentrations than nitrous dioxide [17]. A similar effect was noticed in the case of corona-treated liquid nitrogen (see discussion in [8]). As shown in [17], N_2_O can be produced in reaction $N_{2}\left(A\right)+O\to N_{2}O+O$ that does not require the availability of NO_x_ species. This also results in simultaneous production of ozone:

$$\{N_{2}\left(A\right)+O_{2}\to N_{2}\left(X\right)+O+O\;O+O_{2}+M\to O_{3}+M\;$$

As seen in ozone and nitrous oxide adsorption time profiles (Figure 3b), during the explosion of samples in the presence of air, large amounts of both ozone and N_2_O are being generated. The total area of these profiles indicate that concentrations of these species is almost 5–10 times greater than in the case of evaporation without explosion. We attribute this effect to the significant energy release during the sample decomposition: N≡N triple bond energy is characterized by value of 229 kcal/mol (9.9 eV), while the N=N double and N–N single bond energies are only 100 kcal/mol (4.3 eV) and 38 kcal/mol (1.6 eV), respectively. The highly exothermic conversion of single-bonded nitrogen to diatomic molecular nitrogen could be the source of production of electronically excited $N_{2}\left(A^{3}\Sigma_{u}^{+}\right)$ with the energy of 6.2 eV which leads to generation of N_2_O. We also hypothesize that the registered presence of nitrous oxide and ozone is related to the same processes due to decomposition of material produced by plasma inside of the treatment chamber (because of, for example, plasma-related effects of shock waves, radiation and temperature that trigger the decomposition). It is worth noting that, in the case of evaporation, ozone appears before nitrous oxide due to its lower boiling temperature; at the same time, in the case of explosion, both O_3_ and N_2_O peaks have left shoulder indicating first the evaporation of the existing molecules followed by their fast generation during the explosion.

**Figure 4:**
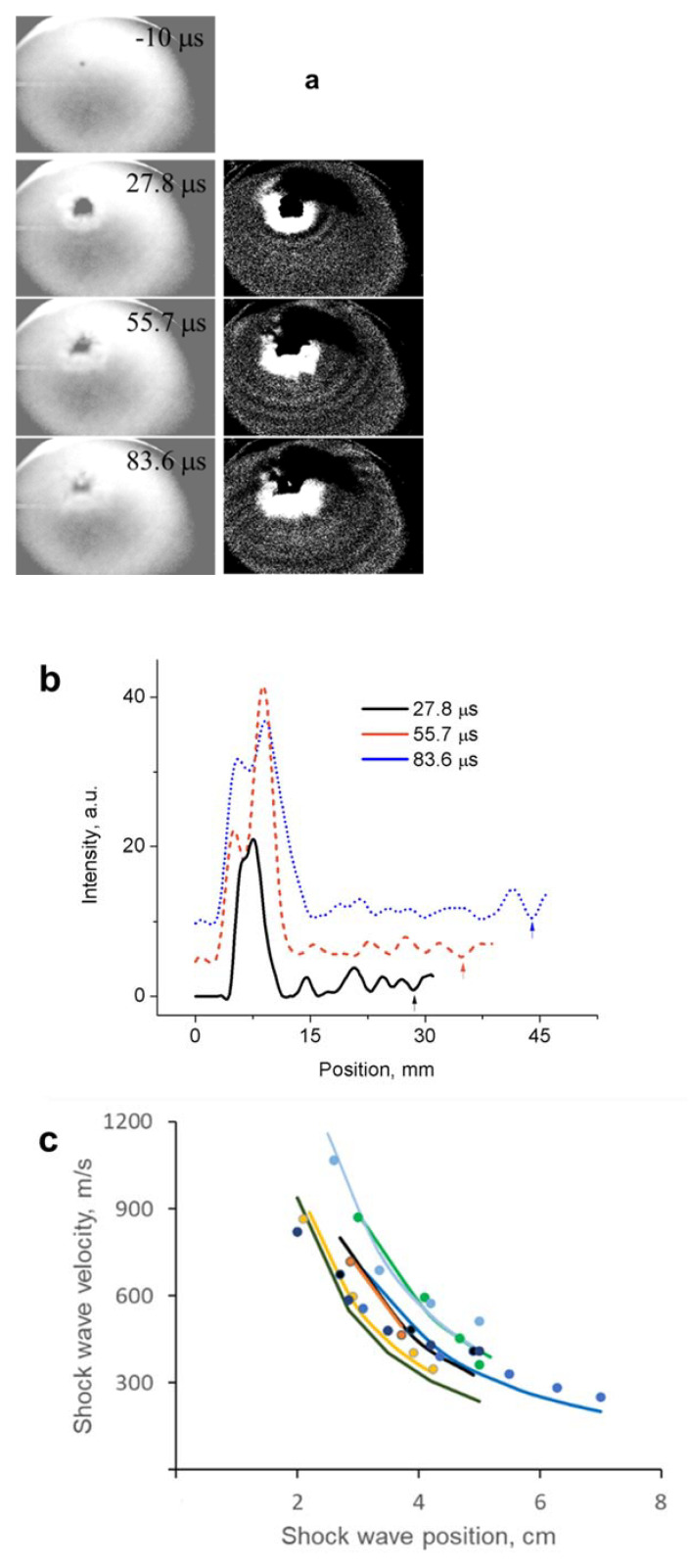
(**a**) Original and ImageJ-enhanced fast-camera captured frames of the explosion video of plasma-treated liquid nitrogen in presence of activated carbon for shock wave imaging. (**b**) Extracted from images above pixel intensity profiles showing position of the shock wave from the center of explosion. (**c**) Measured from the images (markers) and calculated (solid lines) shock wave propagation velocities as a function of the distance from the explosion center for several different experiments.

In order to estimate the energy density of the produced material, we have performed fast shadow imaging of the explosions using an arc lamp as a source of back light and a Phantom Miro M310 high-speed camera. For this, liquid nitrogen was treated together with 50 mg of either crushed activated carbon or Diaion HP-20 for 30 min. After the treatment, from ~100 mL of leftover liquid, several samples of ~20 mL of well-mixed solution were placed in an aluminum dish (diameter of 73 mm) and allowed to settle to room temperature conditions. The explosion event was recorded at rates of 15,000–20,000 fps, and images of the shock wave position were analyzed frame-by-frame using ImageJ software (Figure 4). Using a simplified equation for propagation of a spherical shock wave from a point explosion [18] $D={\left[\frac{3}{4\pi }\times \frac{\left(\gamma -1\right){\left(\gamma +1\right)}^{2}}{3\gamma -1}\right]}^{\frac{1}{2}}{\left(\frac{E}{\rho }\right)}^{\frac{1}{2}}\;R^{-\frac{3}{2}}$, where D is shock wave velocity, R—distance from the center, E—energy of the explosion, ρ = 1.225 kg/m^3^—air density and γ = 1.4—adiabatic index of air, we have estimated the explosion energy of the produced material to be 110 ± 30 J (Figure 4). In a separate set of experiments, using the same treatment conditions and amounts of treated material, the weight of the material was video recorded before and after the explosion in a closed volume (to prevent loss of the adsorbent while generated gases were allowed to escape) using Fisher Scientific Accu-64 analytical balance. Using this technique, we estimate the weight of the exploded material to be 8.2 ± 2.1 mg—this includes all materials that were lost, including ozone, which we estimate to be a few ppm (from the FTIR measurements reported above), or $\sim 10^{-8}$ mg. Therefore, we can estimate total energy density of the material produced by nanosecond-pulsed spark in liquid nitrogen to be about 13.3 ± 3.5 kJ/g, or 3.2 ± 0.8 in TNT (energy density 4.1 kJ/g) equivalent, which is close to the predicted number for polymeric nitrogen network of ~11.3 kJ/g and higher than that for cg-N (9.7 kJ/g) [1]. To compare, the energy density of ozone is only 2.9 kJ/g.

## 4. Conclusions

In this paper, we report on the observation of generation of energetic nitrogen-based material by nanosecond-pulsed spark plasma in liquid nitrogen. Using experimental techniques, we have shown:

1. Nanosecond-pulsed spark plasma treatment of liquid nitrogen results in generation of fluorescent material, which closely resembles the Raman spectra of amorphous nitrogen previously obtained using high pressure compression of molecular nitrogen. The fluorescence disappears at a temperature around −150 °C. The material exhibits unstable properties when heated, but explosions are poorly reproducible.
2. The addition of adsorbents with characteristic pore sizes of >5 nm (indicating that only relatively large molecules are adsorbed) allows the reproducible recovery of the produced material and its explosion upon heating. The fluorescence of the Raman spectra of the physisorbed material completely disappears at around −120 °C.
3. Similar to our previous study with nanosecond-pulsed corona treatment of liquid nitrogen [8], FTIR measurements of the sample decomposition via evaporation compared to its explosion in air reveal the generation of only ozone and nitrous oxide (while no other NO_x_ species were detected), which are believed to be generated via electronically excited nitrogen produced during the polynitrogen decomposition.
4. Shadow imaging of the shock wave propagation from the exploded material allowed us to estimate its energy density to be about 13.3 ± 3.5 kJ/g (3.2 ± 0.8 in TNT equivalent), close to the predicted number for the polymeric nitrogen network of ~11.3 kJ/g.

It should be noted, there is a small but unlikely possibility that the resulting energetic material is related to the erosion of the plasma generating electrode. For example, as shown in [19], potentially metastable Fe-N systems have estimated energy densities of about twice that of TNT.

## Figures and Tables

**Table 1:** Adsorbents used in the study and their characteristics.

Adsorbent	Pore Size, nm	Particle Size, μm
Zeolite (Sigma-Aldrich)	0.1–1	<20
Aluminum oxide, mesoporous (Sigma-Aldrich)	3.8	5.65 μm
Aluminosilicate, mesostructured (Sigma-Aldrich)	2–4	-
AmberSep OPTIPORE L-493 (Supelco)	4.6	300–800
Diaion HP-2MG methacrylic ester copolymer (Supelco)	17	300–750
Diaion HP-20 (Supelco)	26	250–850
Crushed granulated activated carbon	>50	-

## Data Availability

Not applicable.

## References

[1] Eremets M.I., Gavriliuk A.G., Trojan I.A. (2007). Single-crystalline polymeric nitrogen. Appl. Phys. Lett..

[2] Zarko V.E. (2010). Searching for Ways to Create Energetic Materials Based on Polynitrogen Compounds (Review). Combust. Explos. Shock. Waves.

[3] Steele B.A., Stavrou E., Crowhurst J.C., Zaug J.M., Prakapenka V.B., Oleynik I.I. (2017). High-Pressure Synthesis of a Pentazolate Salt. Chem. Mater..

[4] Li Y., Feng X., Liu H., Hao J., Redfern S.A.T., Lei W., Liu D., Ma Y. (2018). Route to high-energy density polymeric nitrogen t-N via He−N compounds. Nat. Commun..

[5] Klapötke T.M., Martin F.A., Stierstorfer J. (2011). C2N14: An Energetic and Highly Sensitive Binary Azidotetrazole. Angew. Chem. Int. Ed..

[6] Eremets M.I., Gavriliuk A.G., Trojan I.A., Dzivenko D.A., Boehler R. (2004). Single-bonded cubic form of nitrogen. Nat. Mater..

[7] Eremets M.I., Eremets R.J., Mao H.-K. (2001). Semiconducting non-molecular nitrogen up to 240 GPa and its low-pressure stability. Nature.

[8] Dobrynin D., Rakhmanov R., Fridman A. (2019). Nanosecond-pulsed discharge in liquid nitrogen: Optical characterization and production of an energetic non-molecular form of nitrogen-rich material. J. Phys. D Appl. Phys..

[9] Dobrynin D., Rakhmanov R., Fridman A. (2019). Nanosecond-pulsed spark discharge plasma in liquid nitrogen: Synthesis of polynitrogen from NaN3. J. Phys. D Appl. Phys..

[10] Touya G., Reess T., Pecastaing L., Gibert A., Domens P. (2006). Development of subsonic electrical discharges in water and measurements of the associated pressure waves. J. Phys. D Appl. Phys..

[11] Šimek M., Pongrác B., Babický V., Člupek M., Lukeš P. (2017). Luminous phase of nanosecond discharge in deionized water: Morphology, propagation velocity and optical emission. Plasma Sources Sci. Technol..

[12] Marinov I., Starikovskaia S., Rousseau A. (2014). Dynamics of plasma evolution in a nanosecond underwater discharge. J. Phys. Appl. Phys..

[13] Pongrác B., Šimek M., Člupek M., Babický V., Lukeš P. (2018). Spectroscopic characteristics of H α /OI atomic lines generated by nanosecond pulsed corona-like discharge in deionized water. J. Phys. D Appl. Phys..

[14] Marinov I., Guaitella O., Rousseau A., Starikovskaia S.M. (2013). Modes of underwater discharge propagation in a series of nanosecond successive pulses. J. Phys. D Appl. Phys..

[15] Dobrynin D., Seepersad Y., Pekker M., Shneider M., Friedman G., Fridman A. (2013). Non-equilibrium nanosecond-pulsed plasma generation in the liquid phase (water, PDMS) without bubbles: Fast imaging, spectroscopy and leader-type model. J. Phys. D Appl. Phys..

[16] Lipp M.J., Park Klepeis J., Baer B.J., Cynn H., Evans W.J., Iota V., Yoo C.-S. (2007). Transformation of molecular nitrogen to nonmolecular phases at megabar pressures by direct laser heating. Phys. Rev. B.

[17] Kossyi I.A., Kostinsky A.Y., Matveyev A.A., Silakov V.P. (1992). Kinetic scheme of the non-equilibrium discharge in nitrogen-oxygen mixtures. Plasma Sources Sci. Technol..

[18] Zel’dovich Y.B., Raizer Y.P. (2002). Physics of Shock Waves and High-Temperature Hydrodynamic Phenomena.

[19] Bykov M., Bykova E., Aprilis G., Glazyrin K., Koemets E., Chuvashova I., Kupenko I., McCammon C., Mezouar M., Prakapenka V. (2018). Fe-N system at high pressure reveals a compound featuring polymeric nitrogen chains. Nat. Commun..

